# Optimization and production of holocellulosic enzyme cocktail from fungi *Aspergillus nidulans* under solid-state fermentation for the production of poly(3-hydroxybutyrate)

**DOI:** 10.1186/s40694-022-00147-6

**Published:** 2022-12-16

**Authors:** Mayur G. Naitam, Govind Singh Tomar, Rajeev Kaushik

**Affiliations:** Division of Microbiology, ICAR-Indian Agricultural Research Institute, New Delhi, 110012 India

**Keywords:** Fungal holocellulase, Fungal enzymes, Xylanase, *Aspergillus nidulans*, Wheat straw, Paddy straw, PHB

## Abstract

The production of petroleum-based plastics increased dramatically following industrialization. Because of multifaceted properties such as durability, thermostability, water resistance, and many others, these plastics have become an indispensable part of daily life. However, while improving people’s quality of life, indiscriminate use of plastics has caused pollution and raised environmental concerns. To address this situation and reduce environmental risks, microbially produced biopolymers such as poly-3-hydroxyalkanoates can be used to make bioplastics that are completely biodegradable under normal environmental conditions. At the moment, the cost of bioplastic production is high when compared to petroleum-based plastics, so alternate strategies for making the bioplastic process economical are urgently needed. Agricultural waste is abundant around the world and can be efficiently used as a low-cost renewable feedstock after pretreatment and enzymatic hydrolysis. Fungi are well known as primary degraders of lignocellulosic waste, and this property was used in the current study to enzymatically hydrolyze the pretreated paddy straw for the production of reducing sugars, which were then used in the microbial fermentation for the production of PHB. In this study, *Aspergillus nidulans* was used to advance a low-cost and efficient enzyme hydrolysis system for the generation of reducing sugars from lignocellulosic biomass. For the production of the holocellulosic enzyme complex, the fungus was grown on wheat straw with Reese mineral medium as a wetting agent. After 216 h of solid-state fermentation at 30 °C, pH 6.0, the enzyme extract from *A. nidulans* demonstrated the highest activity, CMCase 68.58 (± 0.55), FPase 12.0 (± 0.06), Xylanase 27.17 (± 0.83), and β-glucosidase 1.89 (± 0.037). The initial pH, incubation temperature, and time all had a significant impact on final enzyme activity. Enzymatic hydrolysis of pretreated paddy straw produced reducing sugars (8.484 to 30.91 gL^−1^) that were then used to produce poly(3-hydroxybutyrate) using halophilic bacterial isolates. *Burkholderia gladioli* 2S4R1 and *Bacillus cereus* LB7 accumulated 26.80% and 20.47% PHB of the cell dry weight, respectively. This suggests that the holocellulosic enzyme cocktail could play a role in the enzymatic hydrolysis of lignocellulosic materials and the production of PHA from less expensive feedstocks such as agricultural waste.

## Background

The unprecedented increase in industrialization and indiscriminate use of fossil fuels has resulted in severe pollution of the environment and ecosystems. This has created a need for alternative energy and fuel sources, as well as petrochemical-based plastics, which are resistant to degradation and thus accumulate to pollute land and water bodies. Biofuel, bioethanol, biohydrogen, and bioplastics (polyhydroxyalkanoates) can also be produced from inexpensive, renewable, and readily available (in large quantities) feedstock such as lignocellulosic biomass [1, 2]. Lignocellulosic biomass is agricultural crop residue that is produced in large quantities all over the world and can be used efficiently to produce ecofriendly energy compounds [3–5]. Reducing sugars from lignocellulosic waste are used to make bioethanol and bioplastics by various microorganisms, but releasing these reducing sugars from lignocellulosic biomass is difficult because chemical hydrolysis produces toxic compounds that are harmful to the environment and inhibit microbial growth [5–7]. Several fungi and bacteria are known to secrete holocellulosic enzymes capable of degrading lignocellulosic complexes and releasing individual reducing sugars such as glucose, xylose, and arabinose [8]. This provides us with an efficient and cost-effective process for producing fermentable sugars without the production of inhibitory compounds [9]. The holocellulosic enzyme cocktail contains enzymes that work synergistically to degrade cellulose and hemicellulose into individual reducing sugars that are β-1,4 Exoglucanase (EC: 3.2.1.4), β-1,4 Endoglucanase; (EC: 3.2.1.91), β-d-glucosidase (EC: 3.2.1.21), Xylanases i.e. endo-1,4-β-dxylanohydrolase (E.C: 3.2.1.8) [9–11]. Endoglucanase fragments cellulose fibres, releasing free reducing and non-reducing ends [12]. Exoglucanase catalyses the release of small cellobiose oligosaccharides from non-reducing ends. Finally, cellobiose is converted into monomeric sugars by β-glucosidase [13]. Fungi are particularly adept at producing these enzymes and have been commercially used for the development of commercial preparations of these enzymes, as well as being shown to produce such enzymes using various lignocellulosic substrates, such as *Trichoderma reesei* [14], *Aspergillus fumigatus* JCM 10253 [13]*, Trichoderma harzianum* EM0925 [15], *Aspergillus fumigatus* [16], *Aspergillus niger* EFB1 [17], *Trichoderma viride*, *Aspergillus flavus*, *A. niger* and *A. fumigatus* [18]. This economically feasible process can be used to advance future technologies that use lignocellulosic materials as renewable and environmentally friendly resources. Keeping in mind the aforementioned points, the study's goal was to develop a low-cost holocellulosic enzyme cocktail from *A. nidulans* using wheat straw via solid-state fermentation (SSF) to produce fermentable reducing sugars, which will be used in the production of polyhydroxyalkanoates (PHA) from halophilic bacteria using paddy straw as feedstock.

## Results

### Effect of incubation temperature on activity of holocellulosic enzyme extract

**Fig. 1:**
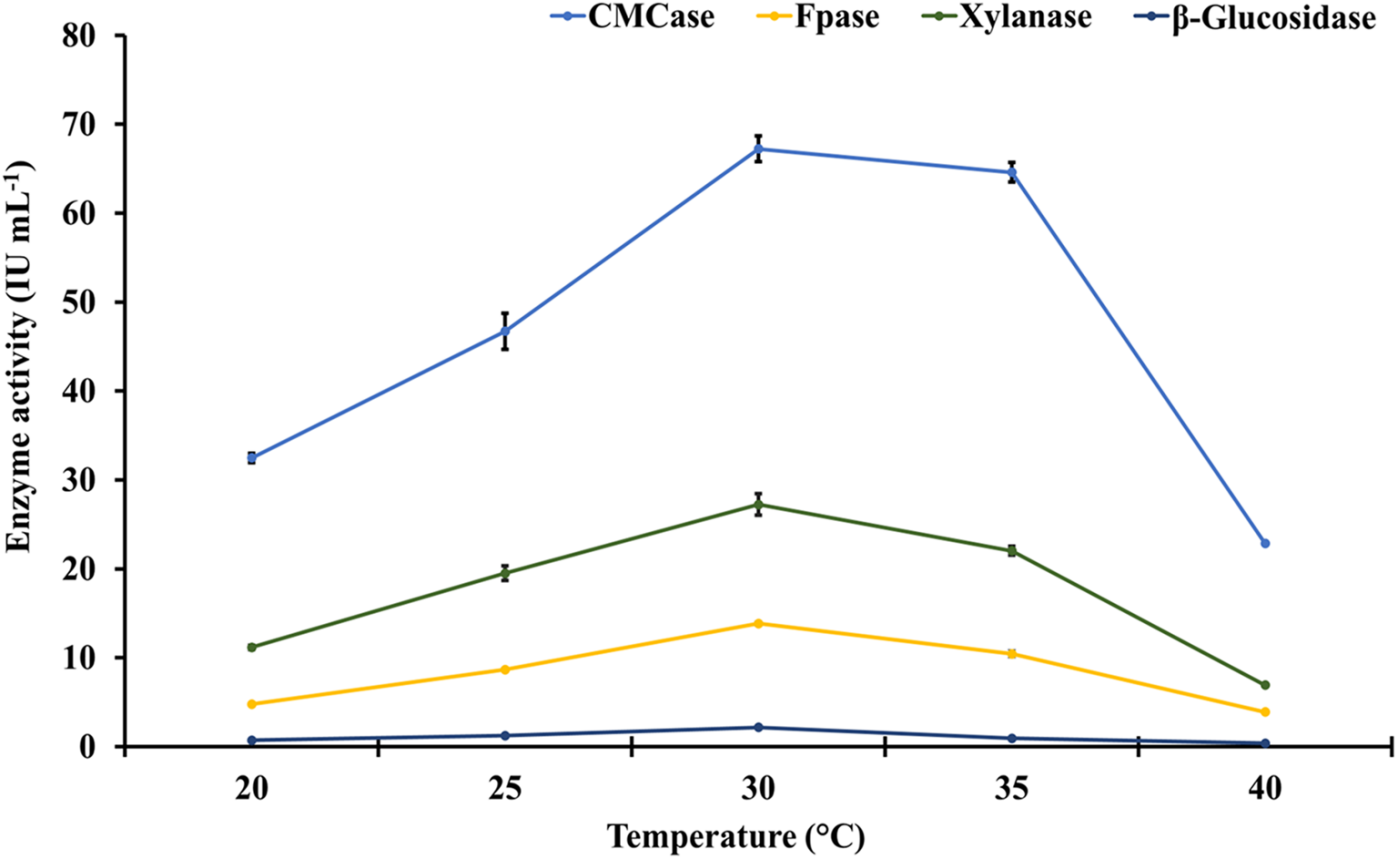
Effect of different incubation temperatures on specific enzyme activity of the crude concentrated holocellulosic enzyme extract from *Aspergillus nidulans.* LSD_p≤0.01_ for (a) temperature: 0.816; (b) enzymes: 0.73; and (c) treatment (a × b): 1.632. The error bar represents the standard deviation

The fungus *A. nidulans* was used to produce a holocellulosic enzyme cocktail using SSF, with wheat straw serving as the substrate for fungal growth. The enzyme activity (IU mL^−1^) of CMCase, FPase, xylanase, and β-glucosidase was measured in the crude concentrated enzyme extract. The crude enzyme extract's activity varied significantly in response to changes in initial pH, incubation temperature, and time. For the fungus to produce maximum growth and enzyme activity, optimal growth conditions are required. Temperature is the most important factor influencing growth and activity, and the results show that temperatures lower or higher than optimal (30 °C) have a significant impact on enzyme activity, resulting in a reduction in enzyme activity of the crude enzyme extract. CMCase, FPase, xylanase, and β-glucosidase all had the highest enzyme activity at 30 °C, with values of 67.23 (± 1.45), 13.84 (± 0.27), 27.23 (± 1.20), and 2.17 (± 0.074) IU mL^−1^, respectively. Lowering the temperature below the optimum resulted in decreased enzyme activity (Fig. 1). Temperatures above the optimum increased the activity of CMCase to some extent but decreased the activity of xylanase and β-glucosidase. At 40 °C, β-glucosidase activity was reduced by 84.48%, while FPase and xylanase activity were reduced by 71.82% and 74.62%, respectively, compared to growth at the optimal 30 °C.

### Effect of pH on activity of holocellulosic enzyme extract

**Fig. 2:**
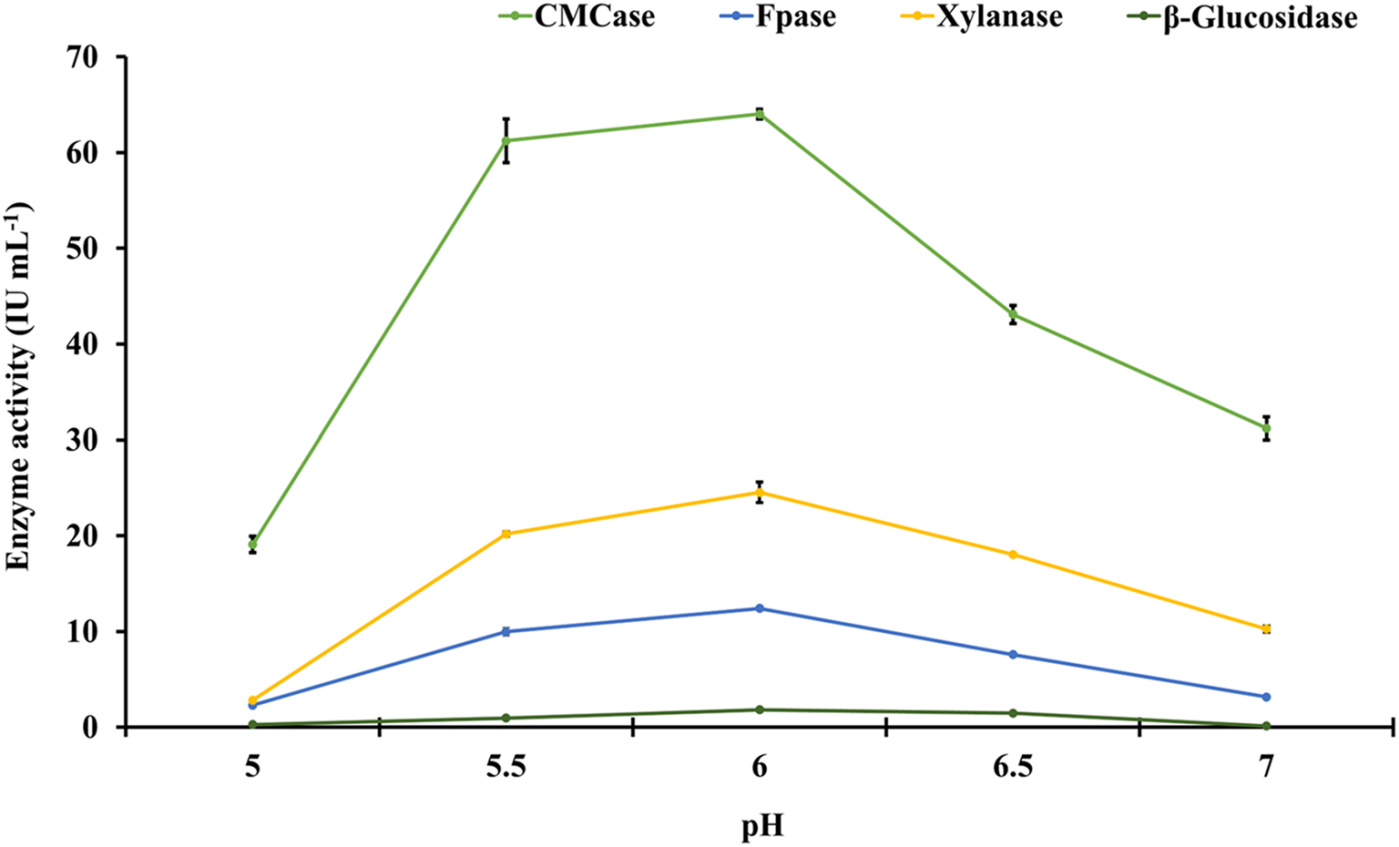
Effect of different initial pH on specific enzyme activity of the crude concentrated holocellulosic enzyme extract from *Aspergillus nidulans.* LSD_p≤0.01_ for (a) pH: 0.805; (b) enzymes: 0.72; and (c) treatment (a × b): 1.61. The error bar represents the standard deviation

The initial pH of the growth medium influences growth because fungi grow better in mildly acidic conditions. Overall enzyme activity was found to be 64.02 (± 0.51), 12.42 (± 0.16), 24.53 (± 1.08), and 1.82 (± 0.04) IU mL^−1^ for CMCase, FPase, xylanase, and β-glucosidase, respectively, when the initial pH of the wheat straw slurry was kept at 6.00. pH values of 6.5 and 5.5 were found to decrease the activity of FPase, xylanase, and β-glucosidase, but not CMCase activity. More acidic conditions (pH 5.0) resulted in a 2.05, 3.93, 2.38, and 15.16-fold decrease in CMCase, FPase, xylanase, and β-glucosidase activity, respectively (Fig. 2). Because of the increased acidity of the wheat slurry, the β-glucosidase activity was the most affected. As a result, the results indicated that any deviation from optimal (both temperature and pH) reduced the enzyme activity of *A. nidulans* crude concentrated enzyme extract.

### Effect of incubation time on activity of holocellulosic enzyme extract

**Fig. 3:**
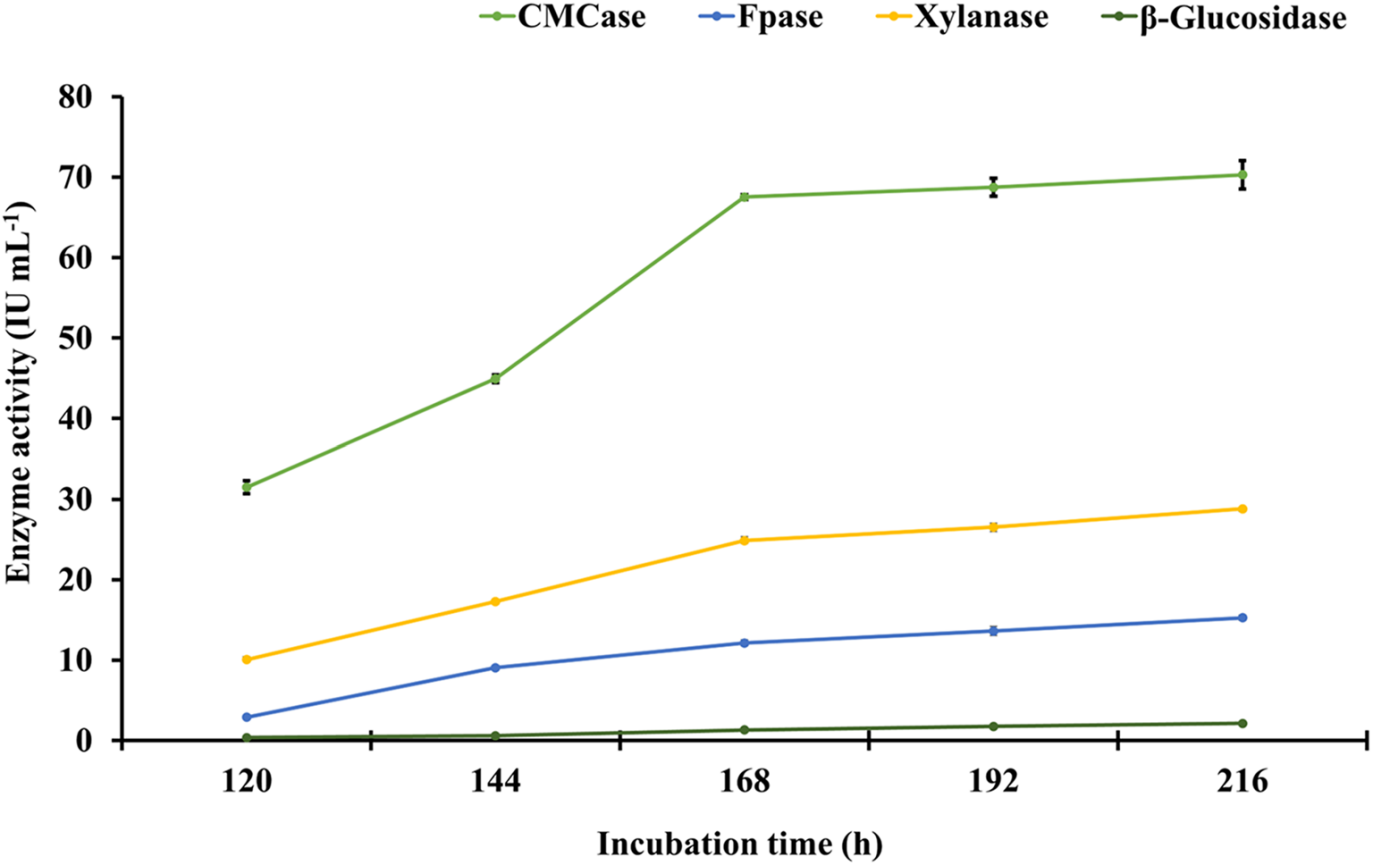
Effect of the different incubation periods on specific enzyme activity of the crude concentrated holocellulosic enzyme extract from *Aspergillus nidulans.* LSD_p≤0.01_ for (a) incubation time: 0.642; (b) enzymes: 0.574; and (c) treatment (a × b): 1.284. The error bar represents the standard deviation

In contrast to ideal temperature and pH requirements, the enzyme activity of the holocellulosic enzyme extract increased invariably with incubation time. The highest enzyme activity for all individual enzyme components was observed at 216 h, i.e., 70.29 (± 1.77) IU mL^−1^ for CMCase, 15.26 (± 0.31) IU mL^−1^ for FPase, 28.81 (± 0.18) IU mL^−1^ for xylanase, and 2.14 (± 0.05) IU mL^−1^ for β-glucosidase, respectively (Fig. 3). However, there was no statistically significant difference in activity observed at 168 h versus a time difference of 48 h (at 216 h). As a result, a 168-h incubation time with enzyme activities of 67.52 (± 0.30), 12.13 (± 0.38), 24.87 (± 0.47), and 1.31 (± 0.04) IU mL^−1^ for CMCase, FPase, xylanase, and β-glucosidase, respectively, was considered optimal for future enzyme production experiments.

Optimum enzyme activity of the holocellulosic crude concentrated enzyme extract from *A. nidulans* was found to be, CMCase 68.58 (± 0.55) IU mL^−1^, FPase 12.0 (± 0.06) IU mL^−1^, xylanase 27.17 (± 0.83) IU mL^−1^ and β-glucosidase 1.89 (± 0.037) IU mL^−1^ when grown using optimum culture conditions as evident from above results i.e. at 30 °C incubation temperature, initial pH 6.0 and incubation time of 7 days. In addition to having significantly higher cellulolytic activity, which can degrade cellulose and release hexose sugars like glucose, the holocellulosic cocktail had significantly higher xylanase activity, which can degrade the hemicellulose complex and release pentose sugars.

### Enzymatic saccharification of the pretreated paddy straw and production of reducing sugars

**Fig. 4:**
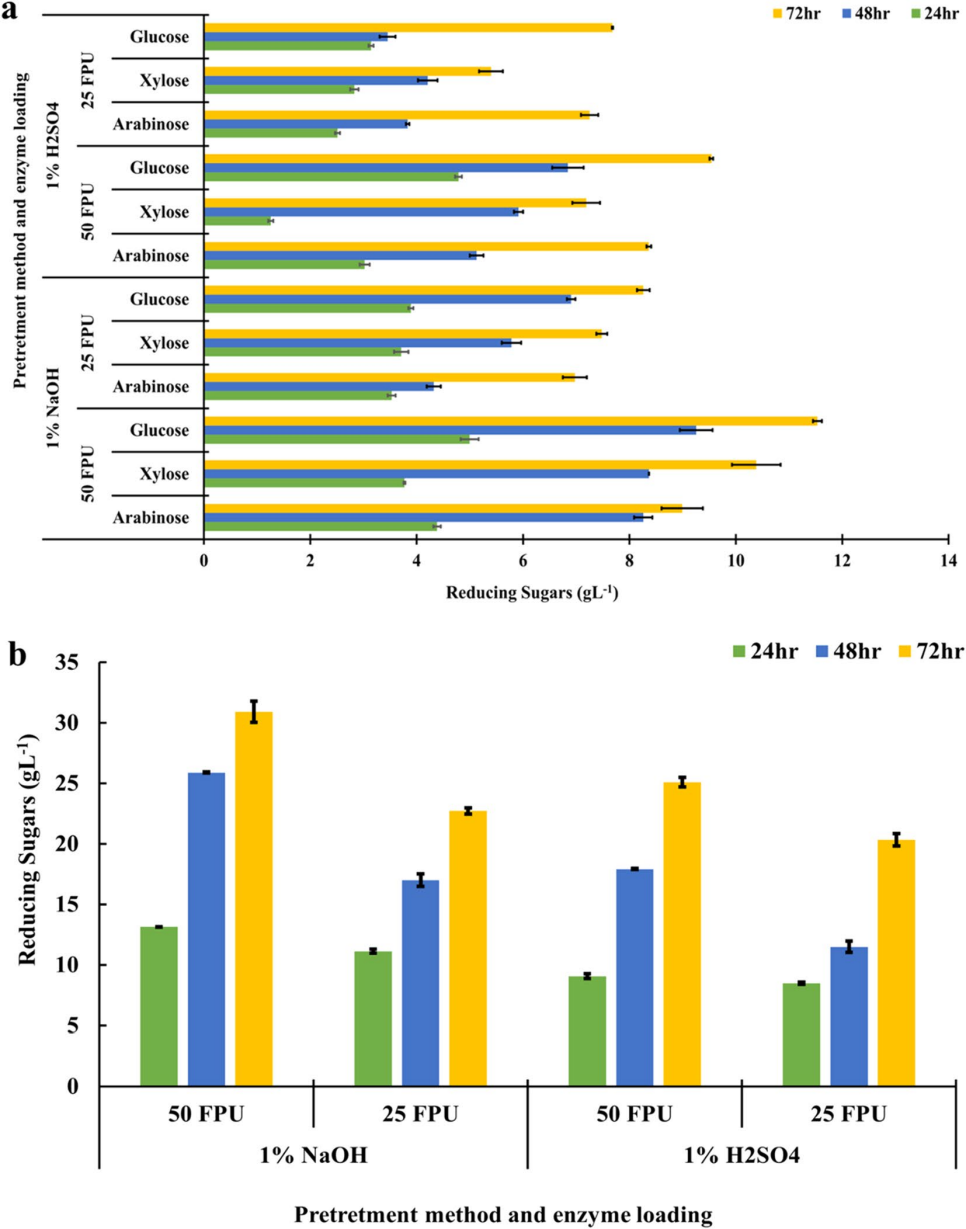
Reducing sugars released (**a** individual sugars, **b** total sugars released) through enzymatic pretreatment using alkali and acid pretreated paddy straw. LSD_p≤0.01_ for (a) pretreatment method: 0.084; enzymes loading: 0.081; incubation time: 0.10; pretreatment method × enzyme loading: 0.116; pretreatment method × incubation time: 0.148; enzyme loading × incubation time: 0.147 and pretreatment method × enzyme loading × incubation time: 0.200. For (b) pretreatment method: 0.372; enzymes loading: 0.369; incubation time: 0.456; pretreatment method × enzyme loading: 0.537; pretreatment method × incubation time: 0.650; enzyme loading × incubation time: 0.652 and pretreatment method × enzyme loading × incubation time: 0.912. The error bar represents the standard deviation

Using *A. nidulans* enzyme extract, alkali and acid pretreated paddy straw was saccharified. Figure 4 depicts the total and individual reducing sugar yield from enzymatic saccharification of pretreated paddy straw. The crude enzymes were tested for saccharification at two different concentrations, 25 and 50 FPU gds^−1^. The percent concentration of total sugars in saccharified paddy straw differed significantly from the control. Alkali-treated paddy straw had a significantly higher amount of total sugars released in the medium than acid-treated paddy straw (Fig. 4). After 72 h of incubation, total sugars were significantly higher in all treatments than after 24 h and 48 h. In acid and alkali-treated paddy straw, the reducing sugar released after fungal holocellulosic enzyme extract-based saccharifications ranged from 8.484 to 30.91 gL^−1^, respectively (Fig. 4). When holocellulosic enzyme extract was used at 50 FPU gds^−1^, the maximum total reducing sugars, 30.91 gL^−1^, were released after 72 h of incubation from alkali pretreated paddy straw. Following that, alkali and acid pretreated paddy straw released 25.88 and 25.09 gL^−1^ reducing sugar after 48 h and 72 h, respectively.

### PHB production using saccharified leachates containing reducing sugars

**Fig. 5:**
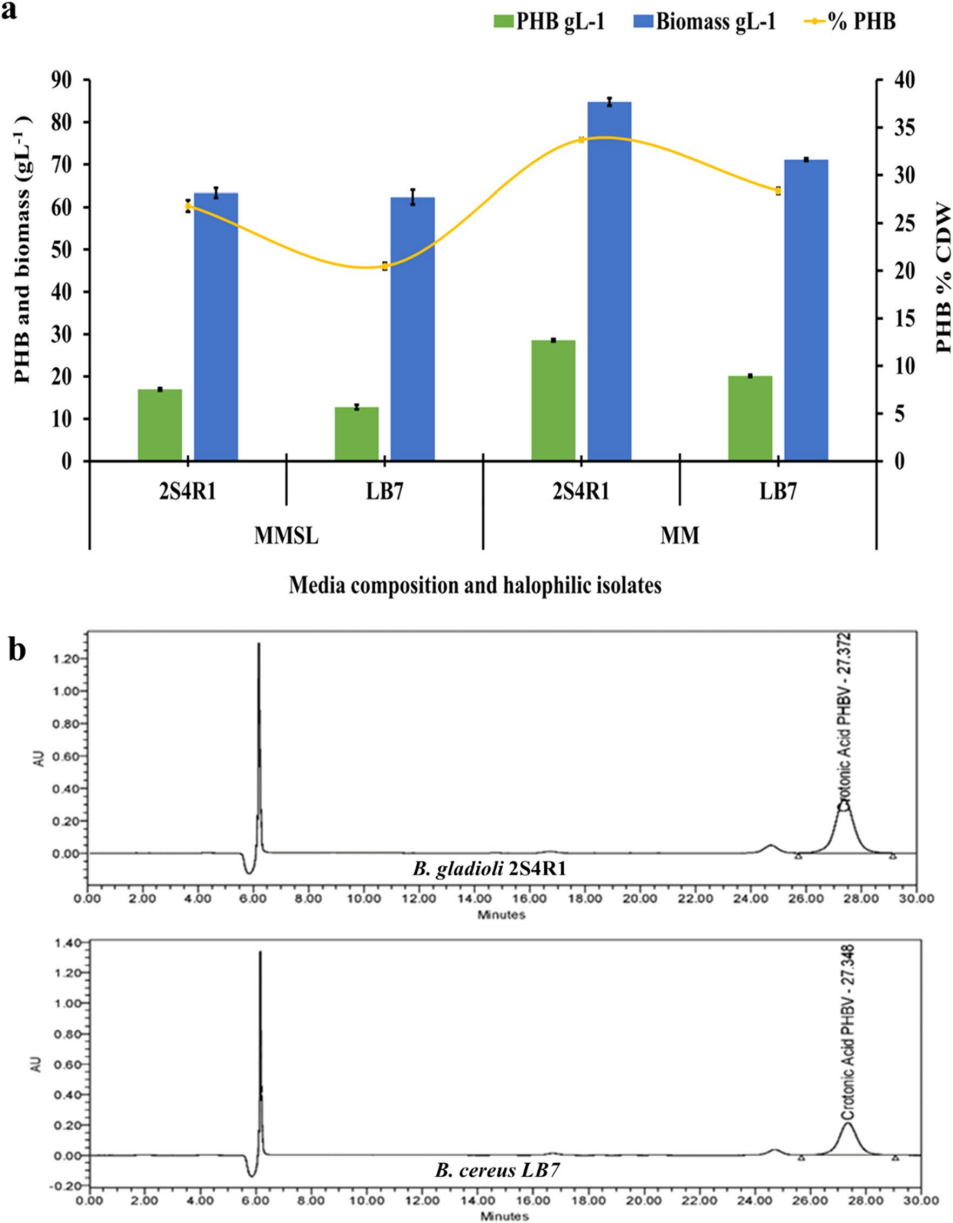
**a** PHB, CDW, and percent PHB production by *B. gladioli* 2S4R1 and *B. cereus* LB7 using MMSL (paddy straw hydrolysates) and MM (glucose) and **b** HPLC quantification of PHB produced by halophilic bacterium 2S4R1 and LB7. LSD_p≤0.01_ for (a) Media formulation: 0.243; bacterial isolates: 0.344; PHB: 0.298; media formulation × bacterial isolates: 0.487; media formulation × PHB: 0.421; bacterial isolates × PHB: 0.596 and media formulation × bacterial isolates × PHB: 0.843. The error bar represents the standard deviation

The growth medium for the isolates *B. gladioli* 2S4R1 and *B. cereus* LB7 for PHB production was developed using saccharified leachate with the highest concentration of reducing sugars (produced by enzymatic hydrolysis of alkali pretreated paddy straw) and was named as Mineral Medium with Saccharified Leachates (MMSL). Because the saccharified leachate contained 30.91 gL^−1^ of total sugars, the C:N ratios of 35:1 and 38:1 for the isolates *B. gladioli* 2S4R1 and *B. cereus* LB7, respectively, were maintained by adjusting the concentration of ammonium sulphate (nitrogen source), with no glucose supplementation. Both isolates, *B. gladioli* 2S4R1 and *B. cereus* LB7, grew well in MMSL, producing 63.35 and 62.34 gL^−1^ biomass and 16.98 and 12.76 gL^−1^ PHB, respectively (Fig. 5a). On a cell dry weight (CDW) basis, the overall PHB content was 26.80% and 20.47%. Mineral medium (MM) with laboratory grade glucose as carbon source showed higher growth and PHB accumulation by isolates *B. gladioli* 2S4R1 and *B. cereus* LB7, with 84.76 and 71.13 gL^−1^ biomass and 33.69 and 28.36% PHB CDW, respectively (Fig. 5b). The PHB accumulation by both the halophilic bacteria was also quantified using HPLC analysis where a retention peak representing crotonic acid was observed near 27 to 28 min (Fig. 5b). The amount of PHB produced was calculated using total area under the peak for the sample and the control (Fig. 5b). *B. gladioli* 2S4R1 and *B. cereus* LB7 were discovered to use sugars generated by enzymatic hydrolysis for intracellular PHB accumulation. As a result, a holocellulosic enzyme cocktail derived from *A. nidulans* via solid-state fermentation may have implications for bacteria-mediated production of biopolymer poly(3-hydroxybutyrate) (PHB).

## Discussion

The fungus *A. nidulans* was used in this study to produce crude holocellulosic enzyme extract via SSF using wheat straw. The activity of the crude holocellulosic enzyme extract from *A. nidulans* was found to be higher than some of the *Aspergillus* species used in the previous studies with a maximum activity of; CMCase 68.58 (± 0.55) IU mL^−1^, FPase 12.0 (± 0.06) IU mL^−1^, xylanase 27.17 (± 0.83) IU mL^−1^ and β-glucosidase 1.89 (± 0.037) IU mL^−1^. A brief account and comparison of cellulolytic enzyme complex and activity from various fungal species have been discussed in Table 1. Using prickly pear as substrate *A. niger* produced an enzyme complex having endoglucanase (4.165 U mL^−1^) and total cellulase activity of 30.293 U mL^−1^ after 70 and 74 h of growth, respectively [19]. Whereas the *Rhizopus* sp. produced an enzyme complex with total cellulase and endoglucanase activity of 13.571 U mL^−1^ and 7.859 using same substrate, respectively [19]. Another species of *Aspergillus* i.e., *Aspergillus* sp. VTM1 was able to produce cellulase enzyme using coffee pulp as carbon source with total enzyme activity of 1.18 U mL^−1^ after 120 h and the optimum pH for the growth and enzyme production was 5.0 [20]. Three fungal isolates i.e., *A. niger, A. terreus and Penicillium* sp. were reported to produce cellulolytic enzymes by Dayal et al. [21]. Among all *A. niger* showed highest endoglucanase and β-glucosidase activity 68.027 U mL^−1^ and 84.74 U mL^−1^, respectively. Significantly higher β-glucosidase activity was observed in *A. terreus* and *Penicillium* sp. than compared to *A. niger*. However, the endoglucanase activity was highest in *A. niger* [21]. Similarly, higher cellulase activity (484.3 U mg^−1^; V_max_ 9.26 U mL^−1^) was observed for enzyme extract produced by fungi *A. niger* using Arachis hypogea shells as substrate for carbon source [22].

**Table 1:** Enzyme activity of holocellulosic enzyme complex produced by various fungal isolates using lignocellulosic biomass as substrate

Organism	Substrate	Enzyme activities				References
		CMCase	FPase	β-Glucosidase	Xylanase	
*Aspergillus nidulans*	Wheat straw	68.58 U mL^−1^	12.0 U mL^−1^	1.89 U mL^−1^	27.17 U mL^−1^	Present study
*Aspergillus niger*	Prickly pear	4.165 U mL^−1^	30.923 U mL^−1^ (total cellulase activity)	–	–	[19]
*Rhizopus* sp*.*	Prickly pear	7.859 U mL^−1^	13.571 U mL^−1^ (total cellulase activity)	–	–	[19]
*Aspergillus* sp. VTM1	Coffee pulp	1.18 U mL^−1^ (total cellulase activity)	–	–	–	[20]
*Aspergillus niger*	–	68.027 U mL^−1^	–	84.74 U mL^−1^	–	[21]
*Aspergillus terreus*	–	50.00 U mL^−1^	–	90.90 U mL^−1^	–	[21]
*Penicillium* sp.	–	62.53 U mL^−1^	–	98.52 U mL^−1^	–	[21]
*Aspergillus niger*	Municipal solid waste	1.19 U mL^−1^	1.504 U mL^−1^	1.39 U mL^−1^	–	[27]
*Trichoderma* sp.	Municipal solid waste	1.95 U mL^−1^	1.77 U mL^−1^	1.66 U mL^−1^	–	[27]
*Trichoderma harzianum* Strain L04	Sugarcane bagasse	4022 U L^−1^	1228 U L^−1^	1968 U L^−1^	–	[28]
*Aspergillus fumigatus*	Sweet sorghum	26 U gds^−1^	3.9 U gds^−1^	6 U gds^−1^	1250 U gds^−1^	[16]

*A. fumigatus* SK1 (54.27 Ug^−1^ CMCase, 3.35 Ug^−1^ FPase, 4.51 Ug^−1^ β-glucosidase and 418.70 Ug^−1^ xylanases) and *A. terreus* M11 (581 Ug^−1^ CMCase, 243 Ug^−1^ FPase, and 128 Ug^−1^ β-glucosidases) were reported to produced enzymes with a significantly higher specific activity using oil palm trunk and corn stover respectively [23, 24]. *A. terreus* M11 has higher cellulolytic enzyme activities than *A. fumigatus* SK1, but it lacks the same level of xylanase activity. Although *A. niger* ATCC 6275 and *A. fumigatus* had higher xylanase activity (282.9 Ug^−1^ and 56.40 Ug^−1^, respectively), their CMCase, FPase, β-glucosidase, and xylanase activities were relatively low [25, 26, 29, 30]. Another *A. fumigatus* strain was found to produce an enzyme cocktail with significantly higher xylanase activity (1250 Ug^−1^ gds) [16]. Additionally, *Trichoderma* sp. is also acknowledged for its use in the production of commercial preparations of the cellulolytic enzyme. Using municipal solid waste (4%) as carbon substrate *Trichoderma* sp. produced 1.77, 1.66 and 1.95 U mL^−1^ exoglucanase, endoglucanase and β-glucosidase, respectively [27], whereas *T. harzianum* strain L04 produced a well-balanced cellulolytic mixture using sugarcane bagasse, i.e. endoglucanase 4022 UL^−1^ (72 h), exoglucanase 1228 UL^−1^ (120 h) and β-glucosidase 1968 UL^−1^ (48 h) under submerged fermentation [28]. The incubation time has a major effect on the activity of individual component enzymes, the activity of endoglucanase was found to be highest at 72 h and that of exoglucanase was at 120 h, whereas only in 48 h, β-glucosidase was observed to have its maximum activity. The effect of different feedstocks on the secretion of the cellulolytic enzyme by *T. reesei* has been discussed in detail by Novy et al. [14].

There is a profound impact of initial pH on cellulosic and hemicellulosic enzyme production. In the present study, the optimum pH for enzyme production was 6.0. A similar correlation was found in the enzyme production by *Aspergillus niger* and *Trichoderma* sp., where 1.76 and 2.16 U mL^−1^, 1.25 and 1.94 U mL^−1^ and 1.44 and 1.71 U mL^−1^ exoglucanase, endoglucanase and β-glucosidase respectively, was produced [27]. Enzyme activity in both fungi was also affected by variation in growth temperature. Being thermophilic, the temperature of 40–50 °C was optimum for production of exoglucanase 1.95 U mL^−1^ and endoglucanase 1.88 U mL^−1^, respectively [27]. *A. fumigatus* was shown to produce maximal activity of endoglucanase at pH 6 and retained residual activity of 57%, 71% and 68% at pH 4, 5 and 7 after 3 h, respectively [16]. In the same study maximum, endoglucanase activity was observed at 55 °C with residual activity of 68%, 71% and 66% retained after incubation for 3 h at 50 °C, 60 °C and 65 °C, respectively [16]. From the results obtained from the present study and discussed above it becomes clear that incubation temperature, time, and initial pH have a strong influence on the final fungal enzyme activity.

Saccharification is critical for the conversion of cellulose into sugars. Because lignin in the cell wall inhibits enzymatic activity, lignocellulosic materials cannot be saccharified by enzymes without pretreatment [29]. A complex enzymatic reaction could be used to perform an enzymatic procedure to hydrolyze cellulosic materials. The cellulolytic catalyst derived from microbial sources provides a one-of-a-kind opportunity for cellulosic matter biodegradation via enzymatic conversion into fermentable sugars [30]. Several reports have been published earlier on enzymatic saccharification of different lignocellulosic biomass [30]. The acid and alkali pretreated paddy straw were used for saccharification by crude enzyme extract from *A. nidulans*. The results of the hydrolysis time effect revealed that the amount of reducing sugars obtained from pretreated paddy straw increased invariably from 24 to 48 h and reached a maximum (30.91 gL^−1^) at 72 h [30]. Liu et al. [31], also reported similar findings, stating that the amount of reducing sugars increased rapidly toward the starting stage (6–72 h), and the most elevated quantity (11.27 gL^−1^) was obtained after 120 h. Among alkali and acid pretreatment, alkali-treated paddy straw was found to have a significantly higher amount of total sugars released in the medium (Fig. 4). Maximum total sugars, 30.91 gL^−1^ (515 mg gds^−1^ at 6% substrate loading), were released after 72 h of incubation from alkali-treated paddy straw @ 50 FPU gds^−1^. Saratale and Oh [32], also preferred alkali pretreated paddy straw over acid pretreated for saccharification and subsequent PHB production. In comparison to acid treatments, alkaline pretreatment was found to be a more effective method for hydrolyzing the complex crosslinking of paddy straw between polysaccharides and lignin [33]. In alkaline pretreatment techniques, the use of high temperature and pressure for hydrolysis of lignin present in agricultural residues is of interest [34]. This type of pretreatment is effective for softwood materials, such as paddy straw, which contain less lignin than hardwood [37–39]. The medium for the growth of isolates *B. gladioli* 2S4R1 and *B. cereus* LB7 for PHB production was formulated using saccharified leachate containing nearly 30 and 25 gL^−1^ of reducing sugars produced during the enzymatic saccharification of alkali and acid pretreated paddy straw with fungal holocellulase @ 50 FPU gds^−1^, respectively.

Fermentation of a hydrolysate containing cellulose, and hemicelluloses is a promising method for converting lignocellulosic biomass into PHA. The total sugar content of paddy straw hydrolysates was found to be between 25.88 and 30.91 gL^−1^. According to Saratale and Oh [32], the hydrolysates must be mostly glucose and xylose with a trace of arabinose. The breakdown and utilization of these sugars by microorganisms is the most important aspect of any powerful fermentation procedure. In this study, we investigated the ability of *B. gladioli* 2S4R1 and *B. cereus* LB7 to produce PHB by utilizing different sugars which were present in the saccharified hydrolysate (MMSL), especially glucose, xylose, and arabinose and compared it with that produced on defined minimal medium (MM) with only glucose as C source. *B. gladioli* 2S4R1 produced significantly higher PHB using MM (33.69% ± 0.30 CDW) and it was statistically significant than the PHB produced using MMSL (26.80% ± 0.60 CDW) (Fig. 5a). In contrast, PHB production by *B. cereus* LB7 in MMSL was not significant (20.47% ± 0.35 CDW) than produced in MM (28.36% ± 0.30 CDW). Cesário et al. [35] fermented wheat hydrolysates containing primarily glucose, xylose, and arabinose using the bacteria *Burkholderia sacchari*. *B. sacchari* was capable of producing 60% PHAs by cell dry weight. Fermentation of commercial hexose and pentose, on the other hand, produced 70% of the CDW of PHAs. *B. firmus* NII 0830 synthesized PHB without detoxification using a pentose stream generated from acid-treated paddy straw, demonstrating its ability to accumulate PHB under stress [36]. The bacterium accumulated 89% PHB on a cell dry weight basis [36]. Rice straw hydrolysates (10.61 gL^−1^ PHB) were found to be a better and more efficient substrate over commercial glucose (8.6 gL^−1^ PHB) for *B. cereus* PS 10 after optimization using response surface methodology (RSM) [37].

Bacterial growth was significantly improved in the current study on media containing paddy straw hydrolysate, indicating the truancy of inhibitors that is common in lignocellulosic biomass. However, various studies have found that detoxifying the hydrolysate before fermentation is necessary to remove inhibitors such as phenolics, furfural, and hydroxymethylfurfural, which may be present in trace amounts [32]. The use of paddy straw hydrolysates resulted in slightly lower PHB production than when using a minimal medium with laboratory-grade glucose (Fig. 5a, b). This significant discovery could have practical implications. The production of PHB by halophilic bacteria from enzymatically hydrolyzed paddy straw has far-reaching implications for agricultural waste biomass management and the development of an economically viable industrial biopolymer production process. The study shows that locally produced fungal holocellulosic enzymes and saccharified leachate extracted from alkali and acid pretreated paddy straw could help reduce the cost of PHB production.

## Conclusion

Various fungi are known for their ability to produce inducible enzymes like cellulases and hemicellulases when grown with substrates like lignocellulosic biomass, food waste, municipal solid waste, and other industrial wastes. These enzymes can be efficiently used for the hydrolysis of lignocellulosic agricultural waste for the generation of fermentable sugars which in turn are important to produce biofuels and bioplastics. In the present study, *Aspergillus nidulans* were used to produce a holocellulosic enzyme cocktail, to develop a low-cost enzyme hydrolysis system for the hydrolysis of paddy straw, which was finally used to produce PHB. The enzyme extract was found to produce, CMCase 68.58 IU mL^−1^, FPase 12 IU mL^−1^, xylanase 27.17 IU mL^−1^ and β-glucosidase 1.89 IU mL^−1^ under solid-state fermentation. Varying growth conditions like incubation temperature and pH were found to affect the enzyme activity of the enzyme extract. Maximum enzyme activity was produced at 30 °C, pH 6.0 and incubation time of 7 days. Enzymatic hydrolysis of paddy straw yielded a significantly higher amount of reducing sugars (8.484 to 30.91 gL^−1^) and employment of *B. gladioli* 2S4R1 and *B. cereus* LB7 resulted in the production of 26.80% and 20.47% of PHB on a cell dry weight basis. Development and use of such a low cost and efficient enzyme hydrolysis and PHB production process will help curb the production cost of PHA, making its industrial production more economic.

## Methods

### Wheat straw as substrate

Wheat straw was collected locally from ICAR-Indian Agricultural Research Institute (IARI) experimental research fields in New Delhi. Straw was collected, washed, and sun-dried for 2 days before being cut into smaller 0.5–1.0 cm pieces and dried in an air-circulation oven for 48 h at 60 °C. Finally, it was ground to a thickness of 10 mm with a grinder and stored for future use.

### Procurement of fungal and bacterial culture and inoculum preparation

The study used a fungal culture of *A. nidulans* that had previously been isolated, characterized, and maintained in the culture collection of the division of Microbiology, ICAR—Indian Agricultural Research Institute (IARI), New Delhi. The fungus was cultured and maintained on Potato Dextrose Agar (PDA) plates supplemented with streptomycin (30 ppm) before being used to make active fungal spore inoculum. Two halophilic bacterial isolates, *B. gladioli* 2S4R1 and *B. cereus* LB7, isolated and screened for PHB production in the past [33, 34] from Rann of Kutch, Gujarat, India, were used in the study to produce PHB from saccharified leachates of pretreated paddy straw. The 16S rDNA sequences of the halophilic bacterial cultures have been submitted to NCBI database with following accession numbers, KY006124.1 and MF370350.1, respectively.

### Production of the hydrolytic enzyme from *Aspergillus nidulans*

Crude holocellulosic enzyme cocktail extract was produced from *A. nidulans* through SSF using finely ground wheat straw. Reese’s mineral medium (RMM) without a carbon source was used as a wetting agent and the solid to liquid ratio was maintained initially at 1:10. Ten grams of finely ground (5–10 mm) wheat straw was taken into a 1000 mL Erlenmeyer flask, and to that 100 mL of RMM was added. The wheat straw and RMM slurry were sterilized via autoclaving at 121 °C for 20 min at 15 psi. Flasks were inoculated with *A. nidulans* culture having a spore count of 10^9^ spores mL^−1^ (measured using hemocytometer) and incubated at 30 °C in a BOD incubator for 9 days. The effect of initial pH, incubation temperature and time was studied by varying the pH (5.0–7.0) with the increment of 0.5, using 0.1 N NaOH and HCL, temperature (20–40 °C) with the increment of 5 °C and time (120–216 h).

### Extraction and concentration of holocellulosic enzyme extract

The crude enzyme cocktail was extracted using a 1:10 sodium citrate buffer by filtration through muslin cloth and centrifugation at 10,000 rpm for 10 min. The filtrate was separated from the pellet, which was discarded. The crude enzyme was concentrated after being precipitated in chilled acetone. The crude enzyme extract was mixed with chilled acetone in a 1:4 ratio and stored at − 20 °C overnight. After centrifuging at 10,000 rpm for 10 min, the supernatant was decanted, and the pellet was suspended in 10 mL of sodium citrate buffer before being centrifuged again at 10,000 rpm for 10 min and discarded. The enzyme concentrate was kept at 4 °C. The crude concentrated enzyme’s enzyme activity was determined and used in subsequent saccharification experiments.

### Quantification of enzyme activity in concentrated crude enzyme

Cellulolytic activities such as Carboxymethyl cellulase (CMCase), Cellobiohydrolase (FPase), xylanases and β-glucosidase were quantified in the concentrated crude enzyme extract.

#### Carboxymethyl cellulase (β-1,4 Endoglucanase; EC 3.2.1.4)

The CMCase activity of the fungal enzyme extract was estimated by incubating the 0.5 mL of holocellulosic enzyme cocktail with 0.5 mL of substrate (0.5 mL of 2% CMC). The substrate was prepared by dissolving 0.2 g of carboxymethyl cellulose in 10 mL of sodium citrate buffer having pH 4.8 and was allowed to dissolve completely by stirring on hot-plate magnetic stirrer (50 °C) for approximately 30 m. [40]. Following incubation, tubes were cooled to room temperature (30 °C) and reducing sugars were quantified following the DNSA reagent method. Three mL of DNSA solution was introduced into the reaction to stop that and kept in a 100 °C water bath for 15 m. Samples were cooled to room temperature (nearly 30 °C) and quantified spectrophotometrically at 575 nm. Finally, the β-1,4 endoglucanase or CMCase activity was expressed as international units (IU) per mL i.e. 1 IU is equal to amount of enzyme able to release 1 µmol of product (reducing sugars) per unit time (per min) during the hydrolysis under assay conditions.

#### Cellobiohydrolase or FPase (β-1,4 Exoglucanase EC 3.2.1.91)

The Exo-β-glucanase activity of the holocellulosic enzyme extract was determined by incubating the enzyme extract (0.5 mL) with 50 mg shredded pieces of filter paper (Whatman No. 1) in 0.5 mL sodium citrate buffer having pH 4.8 for 1 h in 50 °C water bath [40]. After the incubation period, released reducing sugars were quantified spectrophotometrically at 575 nm using DNSA reagent method [41]. The Exo-β-glucanase activity was expressed as filter paper units (FPU) per mL (FPU mL^−1^) as 1 FPU is equal to amount of enzyme releasing 1 µmol of glucose per minute under assay conditions during the hydrolysis. Enzyme and substrate blank, one each were simultaneously run to quantify the natural sugar content of the substrate and enzyme. In enzyme blank, enzyme and citrate buffer, 0.5 mL each (0.05 M) were added. In substrate blank, 50 mg filter paper and 1 mL citrate buffer were added.

#### pNPGase or β-d-glucosidase (EC 3.2.1.21) activity

The β-glucosidase activity of the holocellulosic enzyme extract from the fungus was estimated by allowing 0.5 mL enzyme extract to react with 0.5 mL *p*-nitrophenyl β-d-glucopyranoside for 30 m in a 50 °C water bath [42]. Once incubation was over 1 mL of glycine buffer having pH 10.8 was added to the reaction to end the reaction. The amount of sugars released were quantified spectrophotometrically at 430 nm and the enzyme activity was expressed as µmoles of product (*p*-nitrophenol) released per mL of enzyme extract per minute [42].

#### Xylanases (endo-1,4-β-dxylanohydrolase activity E.C.3.2.1.8)

Xylanases activity was determined as per the method described by Ghose and Bisaria [43] with minor modifications. The reaction mixture with an enzyme consisting of 500 µL of the substrate (birch wood xylan) and the same volume of enzyme filtrate with appropriate dilution was incubated in a 50 °C water bath for 30 m. The amount of xylose released during the reaction was quantified using DNSA reagent method and the endo-1,4-β-dxylanohydrolase enzyme activity of the holocellulosic enzyme extract was expressed as μmoles of xylose sugar released per minute from the hydrolysis reaction [43].

### Pretreatment and saccharification of paddy straw

Five grams of finely chopped and ground paddy straw were pretreated with 1% NaOH and H_2_SO_4_ (alkali and acid pretreatment) for 1 h at room temperature in a static condition and 121 °C for 20 m in an autoclave, respectively. After incubation, the sample was strained with a muslin cloth and washed with distilled water until neutral, dried in an air circulation oven, and stored at room temperature for later use in saccharification. Saccharification of alkali and acid pretreated paddy straw was conducted as per the NREL LAP-009 method by Brown and Torget [44]. Pretreated paddy straw (1.2 g) was administered in 50 mL screw-capped bottles and crude concentrated enzymes derived from *A. nidulans* have been applied @ 25 and 50 FPU g^−1^ Gram Dry Substrate (gds). The FPU activity was used as common currency for referring enzyme activity of fungal holocellulosic enzyme extract in forthcoming experiments. Up to 20 mL of the volume was rendered with a 0.05 M citrate buffer (pH 4.8) and the mixture was incubated in a 50 °C shaker water bath for 72 h. Aliquots from the mixture were sampled at 24, 48 and 72 h, and the amount of reducing sugars released were quantified by HPLC. The HPLC system used in the study was using Waters 515 binary pump and refractive index detector (RID) along with a column oven for maintaining the steady temperature of BIORAD Aminex HPX-87H column at 50 °C throughout the run. 5 mM H_2_SO_4_ was used as mobile phase in the study with a flow rate of 0.5 mL m^−1^ with the help of pump control module and the test samples were sampled for analysis using an autosampler in the HPLC system [45].

### PHB production by selected isolates in media formulated using sugars released by saccharified lignocellulosic biomass

Sugars released during saccharification were used as a carbon source @ 2% based on the sugar content in saccharified products and bacteria were grown on the medium formulated by adding other components of Reese mineral medium. The carbon to nitrogen ratio was adjusted to 30:1 and 38:1 for *B. cereus* LB7 and *B. gladioli* 2S4R1, respectively [38] for the optimum requirement using ammonium sulfate and incubated at 30 °C for 72 h. The minimal media for growing PHB producing isolates were modified by replacing glucose with saccharified leachate and was designated as minimal media with saccharified leachate (MMSL). In case the amount of saccharified sugars is less than 2% in leachate then glucose will be used as a preferred carbon source for supplementing MMSL. Bacterial isolates were grown in MMSL for PHB production and compared with PHB production using standard minimal medium (MM) having laboratory grade glucose as carbon source in triplicates. Based on the growth kinetics studies [38] isolates were incubated at 30 °C for 72 h. PHB from the bacterial biomass was extracted as per the method described by Hahn et al. [46]. Extracted PHB was quantified using HPLC and spectrophotometer as described by Karr et al. [47].

### Statistical analysis

Statistical analysis of the data was performed using online statistical tools of ICAR-Indian Agricultural Statistical Research Institute (IASRI), New Delhi (http://iasri.res.in/analysis/online_analysis.htm) and Origin Pro 2019. Differences were considered unless otherwise indicated, only when relevant at LSD_P≤0.01_.

## Data Availability

The article contains all relevant information. The study’s original contributions are included in the article material; further inquiries can be directed to the corresponding author.
